# Battle for Metals: Regulatory RNAs at the Front Line

**DOI:** 10.3389/fcimb.2022.952948

**Published:** 2022-07-05

**Authors:** Mathilde Charbonnier, Gabriela González-Espinoza, Thomas E. Kehl-Fie, David Lalaouna

**Affiliations:** ^1^ Université de Strasbourg, CNRS, Architecture et Réactivité de l’ARN, UPR9002, Strasbourg, France; ^2^ Department of Microbiology, University of Illinois Urbana-Champaign, Urbana IL, United States; ^3^ Carl R. Woese Institute for Genomic Biology University of Illinois Urbana-Champaign, Urbana IL, United States

**Keywords:** Regulatory RNA, metal ions, metal homeostasis, nutritional immunity, oxidative stress

## Abstract

Metal such as iron, zinc, manganese, and nickel are essential elements for bacteria. These nutrients are required in crucial structural and catalytic roles in biological processes, including precursor biosynthesis, DNA replication, transcription, respiration, and oxidative stress responses. While essential, in excess these nutrients can also be toxic. The immune system leverages both of these facets, to limit bacterial proliferation and combat invaders. Metal binding immune proteins reduce the bioavailability of metals at the infection sites starving intruders, while immune cells intoxicate pathogens by providing metals in excess leading to enzyme mismetallation and/or reactive oxygen species generation. In this dynamic metal environment, maintaining metal homeostasis is a critical process that must be precisely coordinated. To achieve this, bacteria utilize diverse metal uptake and efflux systems controlled by metalloregulatory proteins. Recently, small regulatory RNAs (sRNAs) have been revealed to be critical post-transcriptional regulators, working in conjunction with transcription factors to promote rapid adaptation and to fine-tune bacterial adaptation to metal abundance. In this mini review, we discuss the expanding role for sRNAs in iron homeostasis, but also in orchestrating adaptation to the availability of other metals like manganese and nickel. Furthermore, we describe the sRNA-mediated interdependency between metal homeostasis and oxidative stress responses, and how regulatory networks controlled by sRNAs contribute to survival and virulence.

## Introduction

Trace metals like iron, zinc, manganese, or nickel are essential nutrients for bacteria (Begg, 2019), being cofactors and/or structural components of ~40% of proteins (Andreini et al., 2008). At the same time, these essential nutrients can also be toxic. Since they cannot be synthesized or degraded, bacteria, including pathogens, must adapt to their presence and absence, which is particularly important in the context of infection. During infection, the host renders metals inaccessible to invaders, a process termed “nutritional immunity” (Hood and Skaar, 2012; Nairz et al., 2018; Núñez et al., 2018). This includes extracellular metal withholding through systemic and locally secreted metal-binding proteins such as transferrin, lactoferrin and calprotectin, and metal depletion from phagosomes by host transporters such as NRAMP1. The host also harnesses the toxicity of metals and intoxicates pathogens with high metal levels (Imlay, 2003; Imlay, 2014; Djoko et al., 2015). This mini review will focus on how pathogens adapt to metal limitation and the role of regulatory RNAs in this response and maintaining metal homeostasis. However, it is important to note that the need to adapt changing metal abundance is not restricted to pathogens and that common strategies are used by both pathogenic and environmental microbes.

### Bacterial Countermeasures

Bacteria have evolved multiple mechanisms to maintain metal homeostasis in response to their ever-changing environments. This includes metal importers and exporters, metal storage proteins, and alternative enzymes/pathways to preserve critical enzymatic and metabolic functions (Merchant and Helmann, 2012; Chandrangsu et al., 2017).

Metal import and export systems are critical for bacterial survival and virulence. For instance, in *Staphylococcus aureus*, the uptake of manganese is mediated by the NRAMP homolog MntH and the ABC-type transporter MntABC, while its efflux is carried out by MntE. These systems are crucial not only to maintain manganese homeostasis, but also to resist oxidative stress (Grunenwald et al., 2019; Radin et al., 2019). To efficiently extract metal from their environment, many pathogens also secrete metallophores, molecules having a higher metal-binding affinity than host proteins. Siderophores, iron-chelating secondary metabolites, are important virulence factors used to obtain iron (Kramer et al., 2020; Khasheii et al., 2021). More recently, metallophores that promote zinc uptake and contribute to pathogenesis were identified in *Pseudomonas aeruginosa* and *S. aureus* (Grim et al., 2017; Lhospice et al., 2017). Another approach to obtain these essential nutrients is to steal them from host metal-containing proteins like transferrin, lactoferrin or hemoglobin for iron (Barber and Elde, 2015) and calprotectin for zinc (Stork et al., 2013).

**Figure 1:**
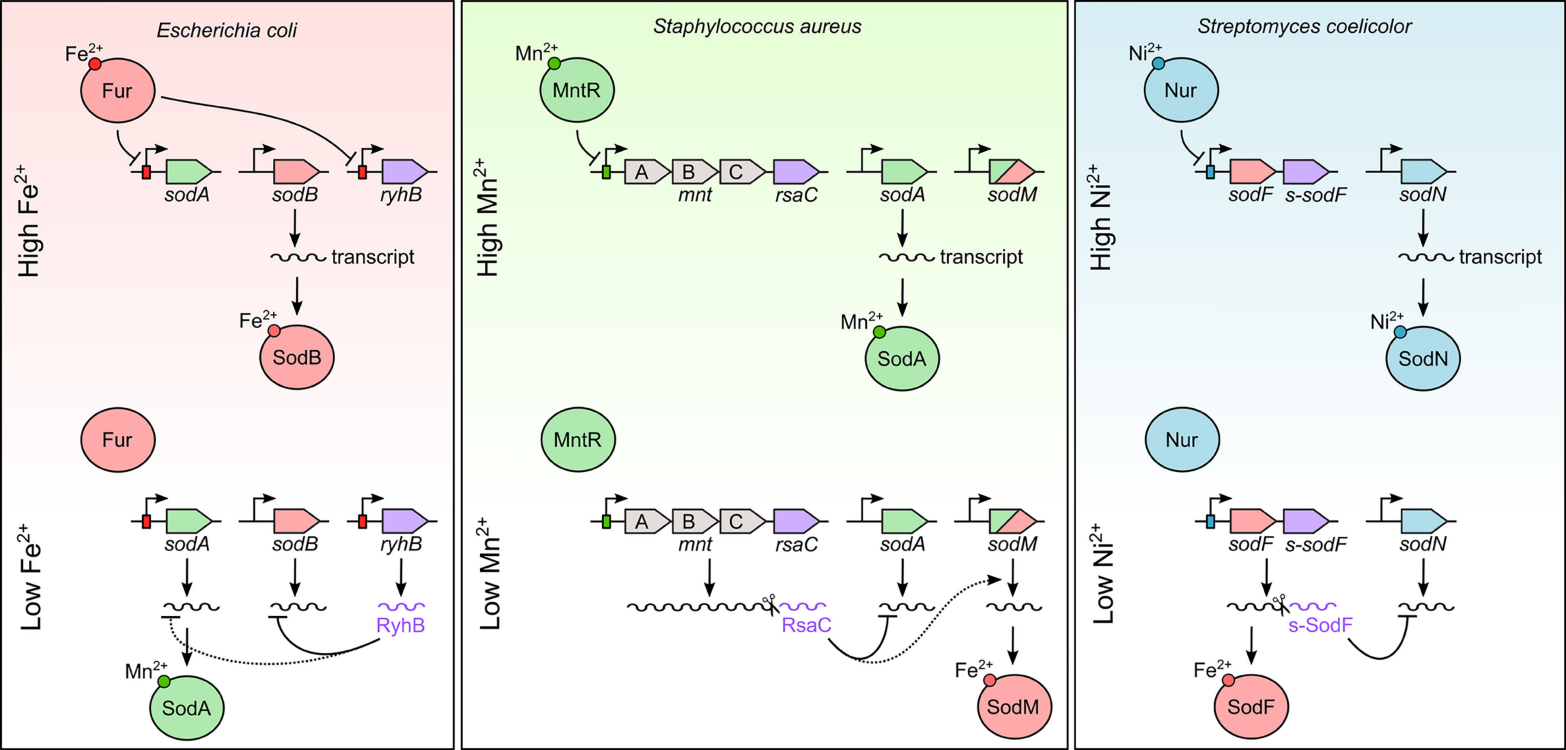
Mixed regulatory circuits between transcription factors and sRNAs to control SOD synthesis according to metal bioavailability. Fur (red), MntR (green) and Nur (blue) boxes are indicated by a rectangle overlapping the promoter. sRNAs are indicated in purple. Dotted lines represent putative or indirect regulation in need of further experimentation. See text for more details.

While important for pathogenesis, the expression of importers is insufficient to enable infection. To cope with metal limitation, bacteria can release iron from storage proteins like ferritin and ferritin-related proteins (Chandrangsu et al., 2017; Bradley et al., 2020). They also alter the composition of the cytoplasm, which buffers metal ions, potentially facilitating metal acquisition by metalloproteins. In response to scarcity, bacteria also reduce their need for the limiting metal, by switching to alternative isozymes or even entire pathways that are not dependent on it (Merchant and Helmann, 2012). A classic example is the use of paralogs of the superoxide dismutase (SOD) associated with distinct metal cofactors to rescue the reactive oxygen species (ROS) detoxification pathway (see below and **Figure 1**).

While beneficial when metal availability is restricted, these adaptations can be deleterious when metals are abundant. Similarly, the adaptations used to overcome metal intoxication, reviewed by Djoko et al. (2015) and Bradley et al. (2020), can be detrimental when metals are limiting. Thus, use of all above-mentioned mechanisms is carefully coordinated by regulators.

## Metal-Responsive Transcription Factors

Classically, the bacterial response to environmental metal abundance is controlled by regulators that directly interact with the target metal. These regulators can be divided into two classes; those that coordinate the response to metal limitation and those that respond to intoxication. Transcription factors that coordinate the response to metal limitation generally repress expression when their target metal is abundant, with converse logic being used to respond to intoxication (Waldron and Robinson, 2009). Metal limitation is frequently sensed by members of either the Fur (Ferric uptake regulator) or DtxR (Diphtheria toxin regulator) family of metal sensing regulators, with divergence enabling the same family to be used to sense different metals (**Table 1**). While primarily negative regulators, positive regulation by these families can occur, for example two MntRs that positively regulate the expression of manganese efflux systems have been reported (Huang et al., 2017; Grunenwald et al., 2019). The Fur family has also been co-opted to coordinate their response to peroxide stress *via* PerR (Bsat et al., 1998; Lee and Helmann, 2006; Pinochet-Barros and Helmann, 2018). In addition, bacteria can leverage protein scaffolds not generally considered metal-responsive to sense metals such as the MarR family proteins, AdcR and ZitR (Varela et al., 2019). While coordinating the response to metal limitation was once thought to be the purview of metal sensing regulators, it is now apparent that metal independent sensors also critically contribute (Nairn et al., 2016; Radin et al., 2016; Harper et al., 2018; Párraga Solórzano et al., 2019; Lonergan et al., 2020). Similarly, there is a growing appreciation for post-transcriptional regulation *via* small regulatory RNAs (sRNAs).

**Table 1:** Transcription factors and sRNAs that respond to metal limitation.

	Metal^a^	Cognate sRNA	Exemplar species
**Fur family^c^**			
Fur	Fe	RyhB and analogs	*E. coli*, *S.* Typhimurium, *P. aeruginosa*, *B. subtilis*...
Zur	Zn	NR	
Mur	Mn	NR	
Nur	Ni	s-SodF	*S. coelicolor*
Irr	Heme	NR	
PerR	Fe, Mn, H_2_O_2_	NR	
**DtxR family^b,d^**			
DtxR	Fe	NR	
MntR	Mn	RsaC	*S. aureus*
**MarR family^e^**			
AdcR/ZitR	Zn	NR	
**Ribbon-helix-helix family^f^**			
NikR	Ni	NikS	*H. pylori*

NR. None reported. ^a^Metal(s) or stimuli that modulate activity in the native organism. ^b^The nomenclature of the DtxR family is heterogeneous with considerable species specificity.

Reviewed by: ^c^Sevilla et al. (2021), ^d^Merchant and Spatafora (2014), ^e^Varela et al. (2019), ^f^Li and Zamble (2009).

## What Are Bacterial Regulatory RNAs?

To enable tighter and fine-tuned regulation, transcription factors are frequently associated with sRNAs into so-called mixed regulatory circuits (Nitzan et al., 2017). Regulatory RNAs in bacteria are generally non-coding and range in size from ~30 to >1,000 nts (Barrientos et al., 2021; Li and Stanton, 2021). Hundreds of sRNAs have been identified in diverse bacteria (Boutet et al., 2022). These critical post-transcriptional regulators control, amongst others, bacterial physiology, stress responses and virulence in response to specific internal or external stimuli such as nutrient availability, oxidative stress, or antibiotics exposure (Chakravarty and Massé, 2019; Mediati et al., 2021).

Regulatory RNAs are mainly categorized in two classes, cis- and trans-encoded. The cis-encoded sRNAs include antisense RNAs and riboswitches that originate from genes located at the same locus as the targeted mRNA. Antisense RNAs are encoded on the opposite DNA strand and, consequently, regulate their cognate mRNA target *via* perfect base-pairings. They are notably involved in toxin-antitoxin systems (Sarpong and Murphy, 2021). Riboswitches are regulatory elements embedded within the 5’ untranslated region (5’UTR) of mRNA targets (Breaker, 2022). Through their aptamer domain, riboswitches sense metabolites or metal ions. Upon ligand recognition, riboswitches are subject to structural modifications which modulate the transcription and/or translation of downstream gene(s).

Trans-encoded sRNAs and their respective targets are located at distant loci. These regulatory RNAs usually regulate multiple targets, from a few to several tens, through imperfect base-pairings (Carrier et al., 2018; Jørgensen et al., 2020). A broad range of regulatory mechanisms are used to control targeted mRNAs positively or negatively. For instance, sRNAs can pair with the Shine-Dalgarno sequence of a specific mRNA, which restricts access to the ribosome binding site and thus blocks mRNA translation. Conversely, sRNAs binding can mask cleavage sites or induce conformational changes promoting mRNA translation. Several RNA-binding proteins, such as Hfq, ProQ and CsrA, play crucial roles in the sRNA-dependent regulation, especially in Gram-negative bacteria. Their roles have been widely discussed in Christopoulou and Granneman (2021) and Quendera et al. (2020).

## sRNAs and Metal Homeostasis

A mere 20 years ago, Massé and Gottesman described the first metal-responsive sRNA, RyhB, which helps reestablish iron homeostasis in starved *E. coli*. Since then, accumulating data revealed that sRNA-mediated adaptive responses to metal fluctuations is not restricted to the model organism *E. coli*, or to iron.

### Iron Homeostasis and RyhB-like sRNAs

RyhB responds to intracellular iron levels as it is directly under the control of Fur (**Table 1**). While Fe^2+^-Fur efficiently represses *ryhB* expression, Fur becomes inactive upon intracellular iron depletion (Masse and Gottesman, 2002). Once produced, RyhB directly interacts with a large set of mRNAs to boost iron import, reduce cellular demands, and redirect iron to essential biological processes (e.g., respiration, DNA synthesis). For more details about this sRNA-dependent iron-sparing response, please refer to Chareyre and Mandin (2018).

Multiple analogs of RyhB have been discovered among bacteria [e.g., PrrF1/2 in *P. aeruginosa* (Wilderman et al., 2004) and FsrA in *Bacillus subtilis* (Gaballa et al., 2008)]. While RyhB-like sRNAs share little to no sequence similarities, they remarkably control a similar set of transcripts and belong to the Fur regulon. In pathogens, RyhB-like sRNAs interlink iron homeostasis and virulence. Porcheron et al. (2014) demonstrated that the deletion of *ryhB* gene in the uropathogenic *E. coli* strain CFT073 leads to a significant reduction of bladder colonization. In *Salmonella* Typhimurium, RyhB-1/2 sRNAs modulate bacterial replication within macrophages and presumably promote immune evasion (Peñaloza et al., 2021). Additional examples are provided by Porcheron and Dozois (2015) and Chareyre and Mandin (2018).

Similar to other adaptations to metal limitation, the synthesis of RyhB sRNA can cause adverse effects on cell physiology and colicin resistance (Lalaouna et al., 2015). Therefore, its production is tightly controlled at both transcriptional and post-transcriptional levels. Lalaouna et al. (2015) identified a tRNA-derived fragment, which protects *E. coli* cells from deleterious effects by “sponging” the transcriptional noise of *ryhB* gene in non-inducing conditions. Other RyhB-like sponging mechanisms have been thereafter described in *E. coli* (AspX; Chen et al., 2021) and in *P. aeruginosa* (SkatA; Han et al., 2016).

### Fur-Independent sRNAs Involved in Iron Homeostasis

In addition to RyhB, several other sRNAs including CsrB/C, CyaR and FnrS have been linked with iron homeostasis.

The RNA-binding protein CsrA and its homologs are pleiotropic post-transcriptional regulators, which control multiple cellular processes including carbon metabolism, stress response, mobility, and virulence (Romeo et al., 1993; Pourciau et al., 2020). In *E. coli*, CsrA also plays a role in iron homeostasis (Pourciau et al., 2019). CsrA reportedly modulates iron storage protein synthesis to remobilize iron during the exponential phase of growth. CsrA also seems to lower RyhB sRNA level *via* an unknown mechanism (Potts et al., 2017). The activity of CsrA is modulated by two sRNAs, named CsrB and CsrC (Liu et al., 1997; Weilbacher et al., 2003), in response to short-chain carboxylic acids and carbon nutritional status (Pannuri et al., 2016; Alvarez et al., 2021). In stark contrast with above-mentioned mechanisms of action, CsrB/C sRNAs bind the CsrA protein *via* recognition motif mimicry, preventing it from interacting with its ‘true’ targets. Noteworthy, both CsrB/C and CsrA are not responsive to iron bioavailability (Pourciau et al., 2019). Hence, CsrA, and by extension, CsrB/C sRNAs could link cellular responses to iron homeostasis notably by integrating additional signals.

Other sRNAs control specific iron-related mRNA targets as recently exemplified by Sy and Tree (2022). The *chuAS* operon encodes a haem receptor and haem oxygenase, involved in haem uptake, and then iron dissociation. This operon is not only regulated by RyhB and Fur in enterohemorrhagic *E. coli*, but also by CyaR and FnrS sRNAs. CyaR activates ChuA translation in response to high cyclic AMP levels, while FnrS sRNA represses ChuS translation during anaerobiosis. Hence, *chuAS* expression is fine-tuned at the infection site by integrating distinct parameters such as oxygen and nutrient bioavailability.

### Manganese-Responsive RNAs

Investigations into metal-responsive RNAs has largely focused on the intersection with iron homeostasis. However, it is now apparent that sRNAs broadly contribute to metal homeostasis. The manganese-responsive sRNA RsaC originates from the 3’UTR of *mntABC* transcript coding for the main manganese ABC transporter in *S. aureus* and is tightly repressed by Mn^2+^-MntR (**Table 1**) (Lalaouna et al., 2019). When *S. aureus* faces manganese starvation, both the MntABC transporter and RsaC sRNA are produced. Lalaouna et al. (2019) demonstrated that, after its release from its precursor through an endonucleolytic cleavage, RsaC modulates the switch between manganese-dependent and iron-utilizing superoxide dismutases (SODs), linking manganese homeostasis to oxidative stress response. More details are provided below and in **Figure 1**. While further investigation is necessary, RsaC could also be a bridge between distinct metal-related networks. In response to manganese limitation, RsaC potentially regulates the transcription factor Zur, the zinc transporter AdcABC, the Fe^3+^-siderophore transporter SstABCD and the iron-sulfur cluster biosynthesis system Suf (Lalaouna et al., 2019).

Regulatory RNAs also contribute to resisting intoxication as the synthesis of bacterial manganese tolerance/efflux systems are controlled by MntR and/or the Mn^2+^-sensing *yybP-ykoY* riboswitch (Dambach et al., 2015; Zeinert et al., 2018; Waters, 2020). Upon Mn^2+^ binding, the *yybP-ykoY* riboswitch either releases the ribosome binding site or forms an antitermination structure allowing the translation or transcription of the downstream gene, respectively. Remarkably, this riboswitch is highly conserved, as over 1,300 bacterial genomes contain one or two *yybP-ykoY* motifs (Zeinert et al., 2018).

### Nickel Homeostasis and Virulence

Regulatory RNAs also contribute to nickel homeostasis and pathogenesis. *Helicobacter pylori*, responsible for benign to severe stomach pathologies, must survive the acidic conditions encountered in the stomach. To that end, *H. pylori* neutralizes acid by producing a nickel-dependent urease, converting urea into ammonia and bicarbonate. The transcription of *ureAB* mRNA encoding the two structural subunits of urease is activated by NikR in presence of Ni^2+^. To prevent advert effects of alkalinization, an antisense RNA to *ureB* is produced in response to elevated pH to lower urease synthesis (Wen et al., 2011; Wen et al., 2013).

More directly connecting nickel-responsive sRNAs and virulence, in 2017, (Vannini et al. (2017) identified three putative NikR-regulated sRNAs. One of these, HPnc4160, renamed NikS, especially controls major virulence and colonization factors including the vacuolating cytotoxin VacA and the carcinogenic protein CagA (Eisenbart et al., 2020; Kinoshita-Daitoku et al., 2021). These examples highlight the role of metal-responsive sRNAs in cell survival and virulence, but also the necessity to integrate multiple environmental signals *via* mixed regulatory circuits to adapt to the host environment.

A conserved metal-sensing riboswitch, named *czcD* and located upstream genes encoding putative cation efflux pumps, has been described as nickel, cobalt and iron-responsive. However, the nature of its ligand is under debate and its physiological role still needs to be clarified *in vivo* (Furukawa et al., 2015; Xu and Cotruvo, 2020).

### The Regulation of Superoxide Dismutases, an Illustrative Example

Superoxide dismutases are ubiquitous enzymes that detoxify superoxide, a toxic compound generated either by aerobic respiration or by immune cells (Abreu and Cabelli, 2010). SODs rely on diverse metal cofactors to function (i.e., manganese, iron, nickel, or zinc/copper). Many bacteria have more than one SOD, each reliant on a different metal cofactor, to cope with fluctuations in metal bioavailability. Regulatory RNAs (RyhB, RsaC and s-SodF) and their cognate transcription factors (Fur, MntR and Nur) (**Figure 1**; **Table 1**) have critical roles in coordinating expression of these multiple SODs in response to metal abundance.

*E. coli* possesses two cytoplasmic (SodA and SodB) and one periplasmic SODs (SodC), which differ in their metal needs and in their temporal regulation. The iron-dependent SodB and the manganese-dependent SodA enzymes are tightly and divergently controlled in response to iron and oxygen abundance (Fee, 1991; Compan and Touati, 1993). As mentioned above, RyhB directly targets several mRNAs coding for non-essential iron-containing proteins such as *sodB* mRNA to spare intracellular iron (Masse and Gottesman, 2002) during iron limitation (**Figure 1**). Under these conditions, the manganese-dependent SOD enzyme, SodA, is induced due to loss of Fe^2+^-Fur mediated repression (Niederhoffer et al., 1990; Compan and Touati, 1993), reestablishing the ROS detoxification pathway. However, RyhB also seems to negatively regulate *sodA* mRNA (Jacques et al., 2006; Argaman et al., 2012). While the molecular rationale for this contrasting regulation is unknown, Semsey (2014) proposed that regulation by both Fur and RyhB allows a faster SodA-dependent response to superoxide stress during iron starvation. This paradoxical regulation of SodA for an sRNA as well studies as RyhB highlights the broader need for continued investigation into the role of sRNAs in metal homeostasis. It should be also noted that there is no evidence that RyhB could control *sodC* mRNA, even in large-scale analyses (Lalaouna et al., 2015; Melamed et al., 2016).

The *S. aureus* genome encodes two SOD enzymes (**Figure 1**), the manganese-dependent SodA and the cambialistic enzyme SodM using either manganese or iron to function (Garcia et al., 2017). As discussed above, the MntR-dependent RsaC sRNA directly blocks SodA synthesis during manganese starvation and facilitates its substitution by the iron-associated SodM (Lalaouna et al., 2019). RsaC likely enables *S. aureus* to spare manganese for essential manganese-containing proteins, avoid the synthesis of a non-functional enzyme and reestablish the ROS detoxification pathway. It is noteworthy that both *rsaC* and *sodM* genes have been acquired exclusively by *S. aureus* and closely related strains, a clear advantage under harsh and selective conditions compared to other staphylococcal species.

SOD regulation also highlights the broad importance of sRNA mediated regulation to non-pathogenic microbes. Indeed, two distinct SODs are encoded in *Streptomyces coelicolor* genome, the nickel-containing SodN and the iron-containing SodF (Kim et al., 2014). These enzymes are antagonistically regulated in response to the cellular level of nickel (Ahn et al., 2006). The nickel-responsive transcriptional regulator of the Fur family, Nur, directly binds to *sodF* promoter and switches its transcription off, while indirectly turns on SodN production (**Figure 1**). Kim et al. (2014) raised the veil on this mystery by identifying s-SodF, an sRNA originating from the 3’UTR of *sodF* mRNA during nickel starvation. This 3’UTR-derived sRNA pairs with *sodN* transcript, induces its degradation and impedes its translation, favoring a nickel-dependent detoxification pathway. Consequently, Nur activates *sodN* transcription *via* the negative regulation of *sodF* transcription.

## Conclusions

Metal-responsive transcription factors and associated sRNAs are involved in intricate regulatory networks, enabling to finely regulate metal homeostasis in response to environmental fluctuations and metal-based immune strategies. This phenomenon is not restricted to *E. coli* and is rather widely distributed among bacteria. These mixed regulatory circuits also play important roles in the virulence and survival of pathogenic strains.

As exemplified above, sRNA-dependent regulations are not limited to iron homeostasis. Manganese and nickel intracellular levels are also tightly regulated *via* trans-encoded RNAs and riboswitches. It is also very likely that other sRNA-dependent metal-sparing responses exist due to the imperative need to balance the import and export of zinc, cobalt or copper. We also highlighted that virulence, metal homeostasis and oxidative stress responses are intimately linked *via* transcription factors and their cognate sRNAs, allowing bacteria to cope with multifactorial environmental fluctuations.

## Author Contributions

MC, GG-E, TK-F and DL contributed to the conceptualization and writing of the manuscript. All authors have read and approved the submitted version.

## Funding

DL and TK-F were supported by Thomas Jefferson Fund, a program of FACE Foundation launched in collaboration with the French Embassy. MC was supported by the “Ecole Doctorale des Sciences de la Vie et de la Santé - ED414”. TK-F was supported by the National Institutes of Health (R01AI155611 and R21AI149115). DL was supported by the "Agence Nationale de la Recherche" (ANR-20-CE12-0021). The work of the Interdisciplinary Thematic Institute IMCBio, as part of the ITI 2021-2028 program of the University of Strasbourg, CNRS and INSERM, was supported by IdEx Unistra (ANR-10-IDEX-0002), by SFRI-STRAT’US project (ANR 20-SFRI-0012), and by EUR IMCBio (IMCBio ANR-17-EURE-0023) under the framework of the French Investments for the Future Program as well as from the previous Labex NetRNA (ANR-10-LABX-0036).

## Conflict of Interest

The authors declare that the research was conducted in the absence of any commercial or financial relationships that could be construed as a potential conflict of interest.

## Publisher’s Note

All claims expressed in this article are solely those of the authors and do not necessarily represent those of their affiliated organizations, or those of the publisher, the editors and the reviewers. Any product that may be evaluated in this article, or claim that may be made by its manufacturer, is not guaranteed or endorsed by the publisher.
